# Postnatal dexamethasone exposure and lung function in adolescents born very prematurely

**DOI:** 10.1371/journal.pone.0237080

**Published:** 2020-08-07

**Authors:** Christopher Harris, Alessandra Bisquera, Sanja Zivanovic, Alan Lunt, Sandy Calvert, Neil Marlow, Janet L. Peacock, Anne Greenough

**Affiliations:** 1 Department of Women and Children’s Health, School of Life Course Sciences, Faculty of Life Sciences and Medicine, King's College London, London, United Kingdom; 2 Asthma UK Centre for Allergic Mechanisms in Asthma, King’s College London, London, United Kingdom; 3 School of Population Health and Environmental Sciences, King’s College London, London, United Kingdom; 4 Department of Child Health, St George's Hospital, London, United Kingdom; 5 EGA Institute for Women’s Health, Faculty of Population Health Sciences, University College London, United Kingdom; 6 NIHR Biomedical Centre at Guy’s and St Thomas NHS Foundation Trust and King’s College London, London, United Kingdom; Centre Hospitalier Universitaire Vaudois, FRANCE

## Abstract

We previously demonstrated corticosteroid administration on the neonatal intensive care unit was associated with reduced lung function at 11 to 14 years of age in children born very prematurely. The objective of this observational study was to assess if lung function remained impaired at 16 to 19 years of age in those who had received postnatal corticosteroids and whether the trajectory of lung function with increasing age differed between those who had and had not received corticosteroids. One hundred and fifty-nine children born prior to 29 weeks of gestational age had comprehensive lung function measurements; 49 had received postnatal dexamethasone. Lung function outcomes were compared between those who had and had not received postnatal dexamethasone after adjustment for neonatal factors. Forced expiratory flow at 75%, 50%, 25% and 25–75% of the expired vital capacity, forced expiratory volume in one second, peak expiratory flow and forced vital capacity and lung volumes (total lung capacity and residual volume) were assessed. The majority of results were significantly lower in those who received dexamethasone (between 0.61 to 0.78 standard deviations). Lung function reduced as the number of courses of dexamethasone increased. Between 11 and 14 years and 16 to 19 years, lung function improved in the unexposed group, but forced expiratory flow at 75% of the expired vital capacity and forced expiratory volume in one second deteriorated in those who had received postnatal corticosteroids (p = 0.0006). These results suggest that prematurely born young people who received postnatal corticosteroids may be at risk of premature onset of chronic obstructive pulmonary disease.

## Introduction

Corticosteroids are frequently given to prematurely born infants to try and reduce the development bronchopulmonary dysplasia (BPD) [1, 2]. Studies in animal models, however, have shown that postnatal corticosteroid administration can alters the lungs development, resulting in delayed alveolarisation and emphysematous lungs with fewer air spaces [3]. We have previously reported that within the United Kingdom Oscillation Study (UKOS) cohort [4], postnatal dexamethasone exposure was associated with poorer respiratory outcomes at two years of age [5] and poorer lung function at 11 to 14 years of age in a dose dependent manner [6].

During puberty there is a period of rapid growth of the airways and lung parenchyma [7]. It is possible that such changes might overcome those differences seen in lung function at 11–14 years. The aim of this study, therefore, was to determine if differences in lung function according to postnatal exposure to corticosteroids persisted at 16 to 19 years of age in very prematurely born young people. In addition, we wished to determine if any lung function changes between 11 to 14 years of age and 16 to 19 years of age differed according to those who did and did not receive postnatal corticosteroids.

## Materials and methods

Children from UKOS were invited to attend for follow-up at 16 to 19 years of age at King’s College Hospital NHS Foundation Trust. Participants and parents gave informed, written consent to take part in the study and ethical approval for the study was given by The North East-Tyne and Wear South Research Ethics Committee.

Forced expiratory flow at 75% (FEF_75_), forced expiratory flow at 50% (FEF_50_), forced expiratory flow at 25% (FEF_25_) and FEF_25-75_, FEV_1_, PEF and forced vital capacity (FVC) were assessed by spirometry. Lung volumes were assessed using a helium-dilution technique (functional residual capacity, FRC_He_) and plethysmography ((FRC_pleth_), total lung capacity (TLC) and residual volume (RV)). Respiratory resistance was measured by impulse oscillometry at 5Hz and 20Hz. The diffusing capacity of the lungs for carbon monoxide was measured using a single breath technique. All tests were done in accordance to guidance from the American Thoracic Society and the European Respiratory Society [8–14]. The results were converted into z-scores to adjust for age, sex, and height [15], except for PEF and respiratory resistance which were expressed as the percentage predicted for height and sex [16, 17]. Lung function was classified as obstructive if FEV_1_/FVC was below the 5^th^ percentile predicted, restrictive if the TLC and FVC were below the 5^th^ percentile predicted, and mixed if the FEV_1_/FVC and TCL were below the 5^th^ percentile predicted [18].

Smoking was assessed by self report and salivary cotinine analysis. Cotinine levels of more than 15 ng/ml were interpreted as active smoking [19]. Pubertal status was ascertained when the participants were aged 11 to 14 years from participant-completed questionnaires using Tanner staging [20, 21].

### Postnatal steroid exposure

Data for postnatal corticosteroid exposure were captured when the original UKOS study was undertaken in 1998 to 2001 [4]. Correspondence confirmed that dexamethasone was the corticosteroid used in the postnatal period in all units. The average course of dexamethasone was 0.25 mg twice daily for three days, followed by 0.15 mg twice daily for three days, followed by 0.05 mg twice daily for three days. In general, repeated courses were considered if the infant remained ventilated in high inspired oxygen concentrations or had been reintubated and weaning was difficult.

### Statistical analysis

Baseline factors were compared between those who were and were not recruited at age 16 to 19 years and those who did and did not receive postnatal dexamethasone (see S1 File). Linear mixed effects models were used to examine the associations between postnatal dexamethasone use, lung function and subsequent exercise capacity. The mixed models allowed for clustering of data arising from multiple births [22] and for confounders. The final models adjusted for”neonatal” factors: sex, mother’s ethnic group, smoking in pregnancy, birthweight z-score, gestational age and oxygen dependency at 36 weeks postmenstrual age (corresponding to moderate to severe bronchopulmonary dysplasia [23]), Apgar score at five minutes, air leak, ventilation group and pulmonary haemorrhage and age at the time of assessment. Measures of dexamethasone use considered were a yes/no indicator and the number of courses of dexamethasone (none, one, two or three). Sensitivity analyses were conducted to explore the robustness of adjustment for confounders. The analyses used propensity score (PS) matching to compare outcomes in children who had and had not been exposed to dexamethasone. PS matching aims to match individuals according to baseline factors to replicate a randomised design. Full details of the PS methodology and the results are given in the supplement (S1 Table in S1 File). Further sensitivity analyses were done to allow for missing lung function data, using multiple imputation. A final sensitivity analysis was done adding antenatal corticosteroids and surfactant into the model. Differences in lung function according to exposure to postnatal corticosteroids, are reported as means and 95% confidence intervals and as proportions with abnormal lung function, below the fifth centile (z-score<-1.64), estimated using the distributional approach [24, 25] (see S1 File). Obstructive, restrictive and mixed abnormal lung function were defined based on z-score cut-offs of FEV_1_/FVC and TLC [15],: obstructive: FEV1/FVC z-score < - 1.645; restrictive: TLC pleth z-score < - 1.645 and a normal FEV_1_/VC (z-score > = -1.645) and mixed: FEV1/FVC z-score < - 1.645 and TLC pleth z-score < - 1.645.

To enable us to determine the trajectory of lung function with increasing age, adjusted linear mixed models were used for each lung function measure with a time-exposure interaction term added along with sex, mother’s ethnic group, smoking in pregnancy, birthweight z-score, gestational age and oxygen dependency at 36 weeks postmenstrual age, Apgar score at five minutes, age at the time of follow-up assessment, air leak, ventilation group, pulmonary haemorrhage and age at the time of assessment as covariates. Comparisons between exposed and non-exposed groups across two follow-up time points (11–14 years and 16–19 years) were carried out for the results of the following measurements: FEF_75_, FEF_50_, FEF_25_, FEV_1_, FVC, FEV_1_/FVC, DLCOc, FRC_pleth_, FRC_He_, and Vcmax. The analyses used all available data between the two time points. Plots of the least squares means with 95% confidence intervals, along with adjusted p-values for the time x exposure interaction are included. Statistical modelling was done using R.

## Results

### Recruitment

**Fig 1:**
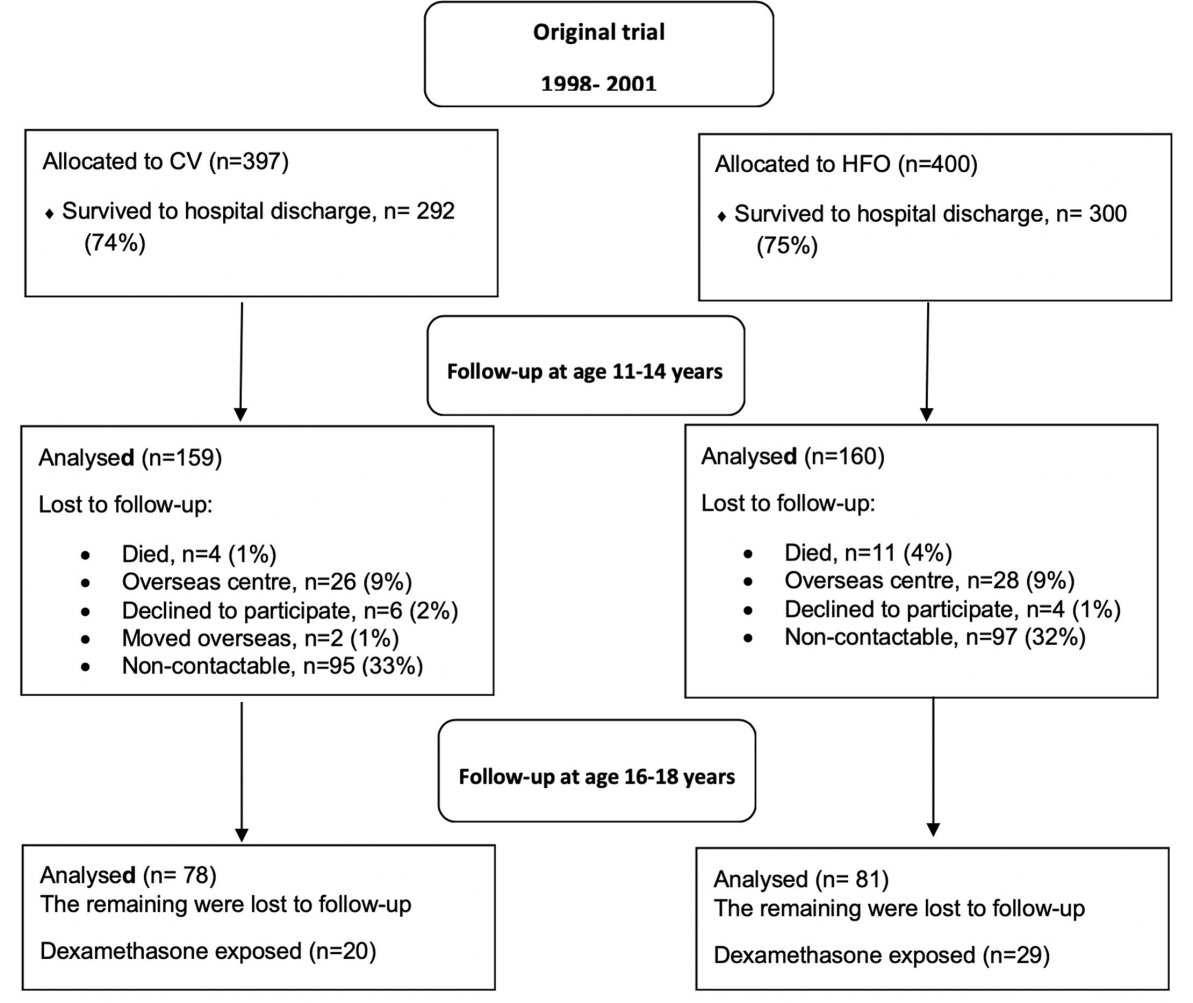
United Kingdom Oscillation Study Consolidated Standards Of Reporting Trials (CONSORT) flow diagram.

Seven hundred and ninety-seven infants were recruited into UKOS from 25 centres; 22 were in England, Scotland or Wales and one in each of Australia, Ireland and Singapore. Three hundred and nineteen were followed up at age 11 to 14 years. Of these, 148 underwent lung function assessment at age 16 to 19 years. A further 11 children who were part of the original trial, but were not followed up at age 11 to 14 years, were followed up at age 16 to 19 years. In total, data from 159 children who completed the lung function assessments at 16–19 years were analysed (Fig 1).

### Baseline characteristics

The participants who were assessed at age 16–19 years had higher mean birthweight and mean gestational age than those not recruited and their mothers were more likely to be white and not to have smoked in pregnancy. They were also more likely to have had a major cranial ultrasound abnormality and pulmonary haemorrhage (S1 Table in S1 File).

Mean follow up at 16 to 19 years was 5.5 years (SD = 0.6) after the 11 to 14 year follow up with no significant difference in the length of follow up between those who received or did not receive postnatal corticosteroids. When seen at 11 to 14 years of age, more than ninety percent of the children had reached at least Tanner stage two. The mean Tanner score for boys and girls for genital or breast development respectively was three. Five percent of boys (3 of 62) and 6% of girls (4 of 69) had reached Tanner stage 5 and finished puberty.

Those who received compared to those who did not receive postnatal dexamethasone were more likely to be male, of lower mean birthweight and gestational age, have a lower Apgar score, have been oxygen dependent at 36 weeks postmenstrual age, more likely to have experienced an air leak or pulmonary haemorrhage and their mothers’ ethnic group was more likely to be white (Table 1).

**Table 1:** Comparison of children who did or did not receive postnatal dexamethasone.

	No dexamethasone	Dexamethasone	P value
N	110	49	
Age	17 (0.8)	18 (0.7)	0.281
Time after 11–14 year follow up (years)	5.44 (0.60)	5.54 (0.58)	0.409
			>
Major cranial ultrasound abnormality*	8 (7)	6 (12)	0.472
Male sex	47 (43)	30 (61)	0.047
Antenatal steroids	99 (90)	45 (94)	0.647
Birthweight (g)	943.4 (217.9)	782.8 (173.1)	<0.001
Birthweight z-score	-0.6 (1.0)	-0.6 (1.2)	0.679
Gestational age, weeks	27.3 (1.3)	25.9 (1.3)	<0.001
Gestational group = 26–28 wk	87 (79)	24 (49)	<0.001
Multiple birth (%)	23 (21)	6 (12)	0.278
Apgar score at 5 mins	8.5 (1.4)	7.3 (2.1)	<0.001
Surfactant given	107 (97)	47 (96)	0.644
Mother smoked during pregnancy	24 (23)	6 (13)	0.222
Mothers ethnic group			0.008
White	91 (83)	48 (98)	
Black	15 (14)	0 (0)	
Other	4 (4)	1 (2)	
Patent ductus arteriosus	25 (23)	19 (39)	0.063
Pulmonary haemorrhage	2 (2)	5 (10)	0.052
Air leak	10 (9)	11 (22)	0.041
Oxygen dependency at 36 weeks postmenstrual age	45 (41)	41 (84)	<0.001
HFOV	52 (47)	29 (59)	0.224
Wheeze (%)	16 (15)	6 (13)	0.949
Antibiotics for chest problems (%)	3 (3)	3 (6)	0.378
Current asthma (%)	10 (9)	4 (9)	>0.999
Inhalers (%)	9 (8)	4 (9)	>0.999
Type of admission (%)			0.092
Chest problems	0 (0)	1 (2)	
Surgery	6 (5)	6 (12)	
Other	0 (0)	0 (0)	
None	104 (95)	42 (86)	
Smoker (%)	11 (10)	3 (6)	0.555

* This outcome was assessed in the early neonatal period, <14 days, as intraventricular haemorrhage with ventricular dilatation or any parenchymal haemorrhage or cystic changes.

The data are presented as the mean (SD) or number (%) unless specified.

### Lung function results

Those who received postnatal corticosteroids had lower mean FEF_75_, FEF_50_, FEF_25_, FEV_1_, FEV_1_/FVC, FEF_25-75_, PEF, FRC_pleth_ and RV_pleth_ z-scores (Table 2). The percentage of children with an abnormal FEF_75_ was 35% in the non-exposed and 60% in the exposed group. The other spirometry measures showed differences between the exposed and unexposed children ranging from 13 to 28 percentage points (Table 3). In the exposed group 60% had an obstructive abnormality compared to 27% in the unexposed group (difference exposed-unexposed 33%, 95% CI 15%, 49%), none of the exposed group had a restrictive abnormality and only 1% of the unexposed group (difference-1%, 95%CI -7%,-6%). There were no young people who had mixed lung function abnormalities.

**Table 2:** Lung function and postnatal dexamethasone exposure.

Lung function	n	No dexamethasone exposure mean (sd) (n = 110)	Dexamethasone exposure mean (sd) (n = 49)	Unadjusted difference (95% CI)	P value	Differences adjusted for neonatal and maternal factors (95% CI)*	P value
FEF_75_ z-score	150	-0.70 (1.14)	-1.81 (1.24)	-1.06 (-1.49, -0.63)	<0.001	-0.72 (-1.23, -0.22)	0.006
FEF_50_ z-score	150	-0.72 (1.05)	-1.57 (0.90)	-0.83 (-1.20, -0.46)	<0.001	-0.70 (-1.15, -0.26)	0.003
FEF_25_ z-score	148	-0.35 (1.11)	-1.28 (0.94)	-0.87 (-1.27, -0.47)	<0.001	-0.81 (-1.30, -0.33)	0.001
FEV_1_ z-score	150	-0.72 (1.28)	-1.74 (1.14)	-0.98 (-1.44, -0.53)	<0.001	-0.65 (-1.19, -0.10)	0.023
FVC z-score	150	-0.15 (1.36)	-0.46 (1.28)	-0.28 (-0.77,0.21)	0.264	-0.01 (-0.62,0.60)	0.984
FEV_1_/FVC z-score	148	-0.89 (1.10)	-1.83 (1.25)	-0.92 (-1.34, -0.50)	<0.001	-0.80 (-1.32, -0.29)	0.003
FEF_25-75_ z-score	150	-1.12 (1.16)	-2.30 (1.22)	-1.13 (-1.56, -0.70)	<0.001	-0.80 (-1.31, -0.29)	0.003
PEF z-score	151	-0.22 (1.05)	-1.08 (1.00)	-0.75 (-1.13, -0.38)	<0.001	-0.73 (-1.21, -0.25)	0.003
DLCOc z-score	149	-1.08 (1.34)	-1.56 (1.16)	-0.47 (-0.94,0.01)	0.055	0.11 (-0.47,0.70)	0.705
DLCOc/VA	149	1.59 (0.21)	1.49 (0.20)	-0.09 (-0.16, -0.01)	0.023	-0.05 (-0.14,0.05)	0.347
TLC_pleth_ z-score	151	0.75 (1.14)	1.13 (1.22)	0.40 (-0.02,0.82)	0.065	0.51 (-0.02,1.04)	0.063
FRC_pleth_ z-score	151	0.39 (1.28)	1.24 (1.39)	0.86 (0.39,1.32)	<0.001	0.78 (0.18,1.37)	0.011
FRC_He_ z-score	128	0.02 (1.34)	0.16 (1.38)	0.14 (-0.38,0.66)	0.599	0.26 (-0.42,0.93)	0.456
RV_pleth_ z-score	150	0.81 (1.18)	1.75 (1.57)	0.94 (0.48,1.40)	<0.001	0.90 (0.32,1.48)	0.003
VC_max_	148	3.88 (1.00)	3.93 (1.13)	0.07 (-0.31,0.45)	0.715	-0.10 (-0.46,0.25)	0.572
LCI	124	9.08 (1.47)	9.89 (1.90)	0.80 (0.18,1.41)	0.012	0.66 (-0.11,1.43)	0.095
R5Hz z-score	155	-0.14 (1.13)	0.07 (1.24)	0.21 (-0.20,0.61)	0.314	0.33 (-0.17,0.83)	0.195
R20Hz z-score	155	0.38 (1.00)	0.29 (1.07)	-0.10 (-0.45,0.26)	0.594	0.10 (-0.34,0.55)	0.650

*adjusted for sex, mother’s ethnic group, birthweight, gestational age, oxygen dependency at 36wk and Apgar score at 5 mins, smoking in pregnancy, age at time of follow-up, air leak, ventilation group, and pulmonary haemorrhage.

**Table 3:** Adjusted differences in mean lung function according to postnatal dexamethasone exposure.

			Estimated adjusted percentage with abnormal lung function		
Lung function	N	**Mean differences adjusted for neonatal and maternal factors (95% CI)**	No dexamethasone exposure	Dexamethasone exposure	Difference (exposed-unexposed) (95% CI)
FEF_75_ z-score	150	-0.72 (-1.23,-0.22)	35%	60%	26% (13%, 38%)
FEF_50_ z-score	150	-0.70 (-1.15,-0.26)	37%	65%	28% (15%, 41%)
FEF_25_ z-score	148	-0.81 (-1.30,-0.33)	22%	50%	28% (15%, 40%)
FEV_1_ z-score	150	-0.65 (-1.19,-0.10)	29%	50%	20% (8%, 33%)
FEV_1_:FVC z-score	148	-0.80 (-1.32,-0.29)	43%	70%	28% (16%, 40%)
FEF_25-75_ z-score	150	-0.80 (-1.31,-0.29)	51%	77%	26% (15%, 37%)
PEF z-score	151	-0.73 (-1.21,-0.25)	16%	38%	22% (11%, 34%)
FRC_pleth_ z-score	151	0.78 (0.18,1.37)	8%	21%	13% (4%, 22%)
RV_pleth_ z-score	150	0.90 (0.32,1.48)	23%	49%	26% (13%, 38%)

* For FRCpleth z-score and RVpleth z-score, an abnormal value is above 5^th^ centile (z>1.645). For all other lung function measures presented here, an abnormal value is taken as below 5^th^ centile (ie z<1.645). The proportion with abnormal lung function was calculated using the distributional approach [25].

Sensitivity analyses were performed on the lung function measurement results with missing outcome and baseline data imputed confirming significant differences between the two groups (S2 Table in S1 File). Propensity score matching on a 1:1 ratio, achieved balance in sex, birthweight, birthweight z-score, gestational age in weeks, smoking in pregnancy, multiple birth, ventilation group and Apgar score and gave effect sizes that were similar to the adjusted difference estimated using mixed models (S3 Table in S1 File). The final sensitivity analyses, with the addition of antenatal corticosteroids and surfactant also had similar effect sizes to the original adjusted model.

The mean lung function (FEF, FEV_1_, FEV_1_/FVC, PEF, FRC_pleth_, RV_pleth_) was lower in children who had received more courses of dexamethasone compared to no courses and this remained significant after adjustment (Table 4).

**Table 4:** Lung function and number of postnatal dexamethasone courses.

		Unadjusted				Differences adjusted* for neonatal and maternal factors			
Lung function	None	One course difference (95% CI)	Two courses difference (95% CI)	Three courses difference (95% CI)	P value	One course difference (95% CI)	Two courses difference (95% CI)	Three courses difference (95% CI)	P value
	N = 110	N = 37	N = 10	N = 2		N = 37	N = 10	N = 2	
FEF_75_ z-score	ref	-0.93 (-1.39,-0.46)	-1.58 (-2.49,-0.68)	-1.95 (-4.27,0.37)	<0.001	-0.63 (-1.15,-0.11)	-1.24 (-2.37,-0.12)	-1.83 (-4.06,0.40)	0.022
FEF_50_ z-score	ref	-0.72 (-1.12,-0.32)	-1.24 (-2.02,-0.47)	-1.64 (-3.63,0.35)	<0.001	-0.63 (-1.09,-0.17)	-1.10 (-2.10,-0.10)	-1.77 (-3.75,0.21)	0.012
FEF_25_ z-score	ref	-0.81 (-1.24,-0.38)	-1.06 (-1.95,-0.17)	-1.77 (-3.89,0.36)	<0.001	-0.76 (-1.27,-0.25)	-0.94 (-2.10,0.21)	-1.89 (-4.05,0.27)	0.011
FEV_1_ z-score	ref	-0.83 (-1.32,-0.34)	-1.41 (-2.36,-0.45)	-3.00 (-5.45,-0.55)	<0.001	-0.54 (-1.11,0.02)	-1.01 (-2.23,0.21)	-2.60 (-5.01,-0.19)	0.041
FVC z-score	ref	-0.19 (-0.71,0.34)	-0.33 (-1.35,0.69)	-3.00 (-5.62,-0.37)	0.137	0.08 (-0.55,0.70)	-0.03 (-1.39,1.32)	-2.49 (-5.17,0.19)	0.328
FEV_1_/FVC z-score	ref	-0.77 (-1.21,-0.32)	-1.76 (-2.69,-0.83)	-0.84 (-3.06,1.39)	<0.001	-0.72 (-1.25,-0.20)	-1.71 (-2.95,-0.47)	-1.01 (-3.26,1.24)	0.009
FEF_25-75_ z-score	ref	-0.97 (-1.44,-0.51)	-1.74 (-2.64,-0.84)	-2.02 (-4.34,0.30)	<0.001	-0.70 (-1.23,-0.18)	-1.38 (-2.51,-0.25)	-1.91 (-4.15,0.33)	0.01
PEF z-score	ref	-0.56 (-0.95,-0.16)	-1.29 (-2.07,-0.52)	-2.14 (-3.55,-0.73)	<0.001	-0.59 (-1.07,-0.11)	-1.29 (-2.32,-0.25)	-2.40 (-3.92,-0.89)	0.002
DLCOc z-score	ref	-0.37 (-0.89,0.15)	-0.86 (-1.81,0.09)	-0.64 (-3.23,1.96)	0.206	0.13 (-0.48,0.74)	0.08 (-1.13,1.30)	-0.57 (-3.33,2.19)	0.941
DLCOc/VA	ref	-0.07 (-0.15,0.02)	-0.15 (-0.30,0.00)	-0.31 (-0.72,0.10)	0.063	-0.04 (-0.14,0.06)	-0.06 (-0.26,0.14)	-0.31 (-0.77,0.14)	0.513
TLC_pleth_ z-score	ref	0.32 (-0.15,0.79)	0.87 (0.01,1.72)	-0.12 (-1.78,1.53)	0.161	0.46 (-0.09,1.01)	1.09 (-0.01,2.19)	-0.07 (-1.79,1.66)	0.158
FRC_pleth_ z-score	ref	0.65 (0.14,1.16)	1.53 (0.59,2.47)	1.51 (-0.32,3.34)	0.001	0.63 (0.02,1.25)	1.48 (0.25,2.71)	1.83 (-0.10,3.76)	0.024
FRC_He_ z-score	ref	0.14 (-0.43,0.71)	0.27 (-0.86,1.39)	-0.59 (-3.27,2.10)	0.894	0.27 (-0.43,0.97)	0.48 (-0.95,1.90)	-0.57 (-3.33,2.18)	0.795
RV_pleth_ z-score	ref	0.64 (0.14,1.14)	1.85 (0.93,2.76)	2.27 (0.48,4.05)	<0.001	0.68 (0.08,1.27)	1.99 (0.80,3.18)	2.46 (0.60,4.32)	0.001
VC_max_	ref	0.17 (-0.24,0.58)	-0.02 (-0.77,0.73)	-1.16 (-2.61,0.30)	0.354	-0.06 (-0.42,0.31)	-0.25 (-0.97,0.48)	-0.63 (-1.77,0.51)	0.68
LCI	ref	0.61 (-0.05,1.27)	1.53 (0.36,2.69)	0.92 (-0.33,2.17)	0.016	0.52 (-0.27,1.32)	1.41 (0.02,2.79)	0.89 (-0.48,2.25)	0.113
R5Hz z-score	ref	0.23 (-0.21,0.67)	0.48 (-0.35,1.31)	-1.31 (-2.92,0.31)	0.182	0.30 (-0.22,0.81)	0.93 (-0.08,1.95)	-0.90 (-2.54,0.74)	0.146
R20Hz z-score	ref	-0.10 (-0.49,0.29)	0.23 (-0.50,0.96)	-1.32 (-2.74,0.10)	0.266	0.03 (-0.43,0.49)	0.88 (-0.03,1.78)	-0.90 (-2.37,0.56)	0.153

*adjusted for sex, mother’s ethnic group, birthweight, gestational age, oxygen dependency at 36wk and Apgar score at 5 mins, smoking in pregnancy, age at time of follow-up, air leak, ventilation group, and pulmonary haemorrhage.

**Fig 2:**
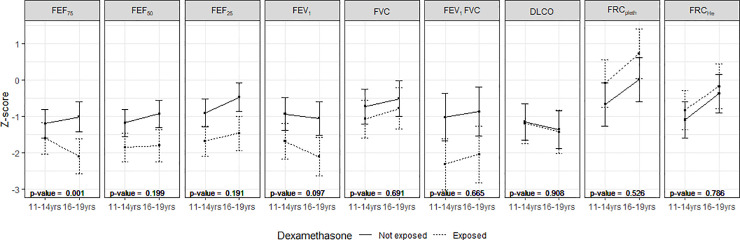
Changes in lung function between 11 and 14 years of age at 16 to 19 years of age. Data are displayed as mean with 95% confidence intervals. Adjusted p-values are given for the time by group interaction showing the difference in the rate of change over time between the two groups.

Comparison of the lung function measures at 11 to 14 years of age to those at 16 to 19 years of age showed an improvement over time in FEF_75_ for those unexposed to postnatal dexamethasone and a deterioration for those exposed (p<0.001) (Fig 2)

## Discussion

We have demonstrated that postnatal corticosteroid administration was associated with significantly poorer lung function in very prematurely born young people aged 16 to 19 years. Those differences remained significant after adjusting for neonatal factors. The differences were substantial, for example in FEF_75_ being equivalent to a difference in 23 percentage points. In addition, the greater the number of courses, the greater the adverse effect on lung function. Importantly, we demonstrate that with regard to FEF_75_ and FEV_1_, there were significant deteriorations in lung function between 11 to 14 years and 16 to 19 years in those who received corticosteroids rather than the improvements in lung function expected during puberty.

Prematurely born young people do not reach the same lung function in early adulthood as do those born at term [26]. Thus, even if their subsequent rate of decline in lung function mirrored that of those born at term, the threshold for respiratory symptoms will be crossed earlier [26]. Amongst children born at term, those who were in the lowest quartile of FEV_1_/FVC at seven years of age were more likely to be at risk of asthma, chronic obstructive pulmonary disease (COPD) overlap syndrome at 45 years of age (odds ratio 16.3, 95% confidence intervals 4.7–55.9) and COPD (odds ratio 5.76, 95% CI 1.9–17.4) [27]. We have demonstrated both at 11 to 14 years [6] and at 16 to 19 years, those who received postnatal corticosteroids had significantly worse lung function than those who did not. We, thus, speculate the postnatal corticosteroid group may be at risk of premature development of COPD. It would, thus, be important to encourage smoking cessation and prevention, maintaining physical fitness, annual influenza immunisation and a general healthy lifestyle [26].

It is recognised that postnatal corticosteroids, particularly if given early i.e. in the first week after birth may increase the risk of neurodevelopmental problems at follow up [1]. We highlight adverse associations on pulmonary outcomes. The young people in this cohort received the commonly then used regimens of postnatal corticosteroids [28], variations of which are still used for infants with severe unresponsive respiratory failure. In view of the adverse effects of systemically administered corticosteroids, lower doses have been used [29] and in infants followed up for two years, no adverse effects were seen [30]. The authors of this study highlighted no definitive evidence of the effect of low dose steroids on morbidity at two years, but highlight that the study did not reach the targeted sample size and was stopped early. Corticosteroids have been also given by inhalation [31]. Although such regimens deliver a lower dose systemically, infants so treated have not been exposed to long term follow up, thus the safety of such regimens cannot be assumed. Research is urgently required to identify safe and effective therapies to prevent chronic oxygen dependency and long-term respiratory morbidity. Furthermore, there is a limited evidence base for treatments of prematurely born infants who remain ventilator and/or chronically oxygen dependent; this should be a further research priority.

Our study has strengths and some limitations. Our study population is similar to those currently receiving neonatal care in that over 90% were exposed to antenatal steroids and postnatal surfactant. They completed a comprehensive range of lung function tests. We attempted to account for confounding factors such as moderate to severe BPD as this has been associated with poorer lung function at follow up [32]. In addition, we do not report the results of a randomised trial, but analysed our data using statistical modelling to control for a variety of other counfounders that is differences in neonatal factors including mode of ventilation. Furthermore the analyses for dexamethasone exposure as a binary outcome were also undertaken using propensity score matching, where cases were matched for their baseline characteristics (S1 Table in S1 File). This analysis also showed significant differences between the two groups. We did not assess pubertal stage at 16–19 years of age follow-up, but all of the children had been assessed at 11 to 14 years of age. The earliest signs of puberty occur at Tanner stage 2 with breast (girls) or genital (boys) development [20, 21]. More than ninety percent of the boys and girls seen at 11–14 years follow up of the UKOS cohort had reached Tanner stage 2. The average time to complete puberty is four to five years [33]. The young people were assessed five years later than their previous respiratory follow up at 11 to 14 years and hence would have been expected to have completed puberty [33]. The mother’s ethnic group was white in eighty seven percent of those who were recruited into this study. The Global Lung Institute reference ranges used in this study do, however, account for ethnicity and hence allow comparison of population derived z-scores across multiple ethnic groups. Twelve of our study population had received more than one course of postnatal steroids. A Cochrane review of late use of postnatal corticosteroids to facilitate extubation demonstrated infants in several studies had more than one course of an open label steroids after randomisation [2]. It is possible, however, that the ongoing severity of such infants’ respiratory problems resulted in their worse lung function at follow-up rather than dexamethasone per se.

In conclusion, we have demonstrated that infants born extremely prematurely who had received postnatal dexamethasone to enhance extubation and reduce the risk of chronic respiratory morbidity had poorer lung function at 16 to 19 years of age in a dose dependent manner. Furthermore, those young people who had received postnatal corticosteroids rather than experiencing the expected increase in lung function between 11 to 14 years of age and 16 to 19 years of age had a deterioration in FEF_75_ and FEV_1_. We suggest those young people may be at risk of premature development of chronic obstructive pulmonary disease. The association of postnatal corticosteroids use and poor lung function would suggest it will be essential to regularly follow them and others exposed to similar regimens worldwide to determine the speed of any subsequent deterioration in lung function and design appropriate interventions to improve their respiratory outcomes.

## Supporting information

S1 File (DOC)
