# Supplementary material for: Postnatal dexamethasone exposure and lung function in adolescents born very prematurely
Source: PLoS One. 2020 Aug 7;15(8):e0237080. doi: 10.1371/journal.pone.0237080 (PMC7413559; doi:10.1371/journal.pone.0237080)
Supplement: S1 File — (DOC) [file pone.0237080.s001.doc]

**Supporting information**

**Postnatal dexamethasone exposure and lung function in adolescents born very prematurely**

Christopher Harris, Alessandra Bisquera, Sanja Zivanovic, Alan Lunt, Sandy Calvert, Neil Marlow, Janet L Peacock, Anne Greenough

**List of tables in supporting information**

**Table S1:** Comparison of baseline characteristics between children recruited or not recruited

**Table S2:** Multiple imputation on missing lung function and covariate data

**Table S3:** Comparison between mean differences in lung function by steroid exposure: unadjusted, adjusted using mixed effects modelling and adjusted using propensity score matching

**Postnatal steroid exposure**

Data for postnatal corticosteroid exposure were captured when the original UKOS study was undertaken in 1998 to 2001 [S1] Correspondence confirmed that dexamethasone was the corticosteroid used in the postnatal period in all units. The average course of dexamethasone was 0.25 mg twice daily for three days, followed by 0.15 mg twice daily for three days, followed by 0.05 mg twice daily for three days.

**Statistical analysis**

**Mixed effects models**

In the mixed effects modelling, the participant was included as a random effect to take account of the relatively high proportion of multiple births [S2]. All other predictor variables were forced into the model and treated as fixed effects. Measures of dexamethasone use considered were: a yes/no indicator and the number of courses of dexamethasone (none, one, two or three). Initially associations were explored in univariable models and then in a multivariable model which adjusted for the baseline covariates that were shown to differ between the exposed and non-exposed groups. Differences in mean lung function results by dexamethasone exposure were also shown as the difference in the proportions with abnormal lung function using the fifth centile in healthy children to define abnormal [S3, S4]. The following factors were included as confounders: sex, mother’s ethnic group, birthweight, gestational age and oxygen dependency at 36 weeks postmenstrual age, Apgar score at five minutes, air leak, ventilation group and pulmonary haemorrhage and age at the time of assessment.

**Propensity Score (PS) matching**

Propensity score (PS) matching works by matching the subjects as closely as possible using baseline factors prior to analysis so that the study closely resembles a randomised trial. The following baseline factors were used in the propensity score matching: sex, birthweight, birthweight z-score, gestational age in weeks, smoking in pregnancy, multiple birth, ventilation group and Apgar score. It is not recommended to adjust for non-baseline factors and so age at assessment was not used in the PS analysis. The main challenge of the PS method is to obtain close matches for all subjects. It was not possible to use propensity score matching for three measures of dexamethasone exposure, that is timing of administration, number of courses and days of exposure due to the small numbers in the different dexamethasone-use categories. For this reason only adjustment by mixed effects modelling was undertaken for those measures.

**Multiple imputation**

Some children were unable to complete all lung function tests and, so, as an additional sensitivity test we used multiple imputation using chained equations to impute missing lung function data as in our previous work [S5]. All outcome data as well as the baseline covariates, sex, mother’s ethnic group, birthweight, gestational age and oxygen dependency at 36 weeks postmenstrual age, Apgar score at five minutes, antenatal steroids, air leak, ventilation group, ultrasound abnormality, patent ductus arteriosus, and pulmonary haemorrhage, were used in the imputation. Fifty data sets were imputed, and the imputation assumed that given these covariates, the data were missing at random.

**Estimating the proportion with abnormal lung function**

In addition to reporting the differences in mean lung function by postnatal steroid exposure, we estimated the proportion in each group with abnormal lung function as a measure of those at high risk. This estimation was done using the fifth percentile as the cut-off for ‘normal’ and estimates the proportion below this using a published statistical method, the distributional approach [S3, S4].

**Results**

Table S1: Comparison of baseline characteristics between children recruited or not recruited.

The data are presented as the mean (SD) or number (%) unless specified

|  | **Recruited** | **Not recruited** | **p** |
| --- | --- | --- | --- |
| **N** | 159 | 638 |  |
| Male | 77 (48) | 351 (55) | 0.161 |
| Mother’s ethnic group |  |  | <0.001 |
| White | 139 (87) | 497 (78) |  |
| Black | 15 (9) | 55 (9) |  |
| Other | 5 (3) | 83 (13) |  |
| Birthweight (g) | 893.9 (217.7) | 843.4 (217.9) | 0.009 |
| Birthweight z-score | -0.6 (1.0) | -0.6 (1.0) | 0.860 |
| Gestational age, weeks | 26.9 (1.5) | 26.4 (1.5) | <0.001 |
| Gestational group = 26-28 wk | 111 (70) | 402 (63) | 0.131 |
| Multiple birth | 29 (18) | 161 (25) | 0.080 |
| Surfactant given | 154 (97) | 615 (96) | >0.999 |
| Mother smoked during pregnancy | 30 (20) | 170 (29) | 0.034 |
| Systemic corticosteroids given prior to extubation | 49 (31) | 150 (29) | 0.695 |
| Oxygen dependency at 36 weeks postmenstrual age | 86 (54) | 257 (57) | 0.552 |
| Oxygen dependency at 28 days | 122 (77) | 384 (82) | 0.176 |
| Oxygen dependent at discharge | 37 (24) | 85 (20) | 0.367 |
| Major cranial ultrasound abnormality | 14 (9) | 115 (18) | 0.005 |
| HFOV | 81 (51) | 319 (50) | 0.901 |
| Patent ductus arteriosus | 44 (28) | 222 (35) | 0.110 |
| Pulmonary haemorrhage | 7 (4) | 92 (15) | 0.001 |
| Air leak | 21 (13) | 115 (18) | 0.185 |

**T**able S2: Multiple imputation on missing lung function and covariate data

| Lung function | n | **Differences adjusted for neonatal and maternal factors without imputation (95% CI)** | **P value** | **Differences adjusted for neonatal and maternal factors with imputed data**  **(95% CI)** | **P value** |
| --- | --- | --- | --- | --- | --- |
| FEF_75_ z-score | 150 | -0.72 (-1.23, -0.22) | 0.006 | -0.59 (-1.04, -0.13) | 0.012 |
| FEF_50_ z-score | 150 | -0.70 (-1.15, -0.26) | 0.003 | -0.61 (-1.01, -0.21) | 0.003 |
| FEF_25_ z-score | 148 | -0.81 (-1.30, -0.33) | 0.001 | -0.74 (-1.19, -0.29) | 0.002 |
| FEV_1_ z-score | 150 | -0.65 (-1.19, -0.10) | 0.023 | -0.54 (-1.03, -0.04) | 0.036 |
| FVC z-score | 150 | -0.01 (-0.62, 0.60) | 0.984 | 0.03 (-0.51, 0.58) | 0.912 |
| FEV_1_/FVC z-score | 148 | -0.80 (-1.32, -0.29) | 0.003 | -0.67 (-1.13, -0.21) | 0.005 |
| FEF_25-75_ z-score | 150 | -0.80 (-1.31, -0.29) | 0.003 | -0.69 (-1.14, -0.23) | 0.004 |
| PEF z-score | 151 | -0.73 (-1.21, -0.25) | 0.003 | -0.70 (-1.12, -0.27) | 0.002 |
| DLCO z-score | 149 | 0.11 (-0.47, 0.70) | 0.705 | -0.06 (-0.58, 0.46) | 0.817 |
| DLCO/VA | 149 | -0.05 (-0.14, 0.05) | 0.347 | -0.06 (-0.14, 0.03) | 0.209 |
| TLC_pleth_ z-score | 151 | 0.51 (-0.02, 1.04) | 0.063 | 0.32 (-0.16, 0.80) | 0.188 |
| FRC_pleth_ z-score | 151 | 0.78 (0.18, 1.37) | 0.011 | 0.64 (0.10, 1.18) | 0.023 |
| FRC_He_ z-score | 128 | 0.26 (-0.42, 0.93) | 0.456 | 0.27 (-0.30, 0.84) | 0.351 |
| RV_pleth_ z-score | 150 | 0.90 (0.32, 1.48) | 0.003 | 0.63 (0.09, 1.18) | 0.025 |
| VC_max_ | 148 | -0.10 (-0.46, 0.25) | 0.572 | -0.03 (-0.35, 0.29) | 0.848 |
| LCI1 | 124 | 0.66 (-0.11, 1.43) | 0.095 | 0.39 (-0.32, 1.11) | 0.285 |
| R5Hz z-score | 155 | 0.33 (-0.17, 0.83) | 0.195 | 0.22 (-0.25, 0.69) | 0.359 |
| R20Hz z-score | 155 | 0.10 (-0.34, 0.55) | 0.650 | 0.09 (-0.31, 0.50) | 0.655 |

**Table S3: Comparison between mean differences in lung function by steroid exposure: unadjusted, adjusted using mixed effects modelling and adjusted using propensity score matching**

| **Lung function** | **Unadjusted mean difference**  **(95% CI)** | **Mean differences adjusted for neonatal and maternal factors (95% CI)*** | **p** | **Mean differences adjusted for neonatal and maternal factors, with the addition of antenatal steroids and surfactant (95% CI)**** | **p** | **Mean differences adjusted using propensity score matching (1:1 ratio)***** | **p** |
| --- | --- | --- | --- | --- | --- | --- | --- |
| FEF_75_ z-score | -1.06 (-1.49, -0.63) | -0.72 (-1.23, -0.22) | 0.006 | -0.76 (-1.27, -0.25) | 0.004 | -0.90 (-1.42, -0.38) | 0.001 |
| FEF_50_ z-score | -0.83 (-1.20, -0.46) | -0.70 (-1.15, -0.26) | 0.003 | -0.70 (-1.15, -0.24) | 0.003 | -0.79 (-1.20, -0.38) | <0.001 |
| FEF_25_ z-score | -0.87 (-1.27, -0.47) | -0.81 (-1.30, -0.33) | 0.001 | -0.79 (-1.29, -0.30) | 0.002 | -0.98 (-1.45, -0.52) | <0.001 |
| FEV_1_ z-score | -0.98 (-1.44, -0.53) | -0.65 (-1.19, -0.10) | 0.023 | -0.64 (-1.20, -0.08) | 0.027 | -0.94 (-1.42, -0.46) | <0.001 |
| FVC z-score | -0.28 (-0.77, 0.21) | -0.01 (-0.62, 0.60) | 0.984 | 0.02 (-0.60, 0.64) | 0.956 | -0.30 (-0.84, 0.24) | 0.284 |
| FEV_1_/FVC z-score | -0.92 (-1.34, -0.50) | -0.80 (-1.32, - 0.29) | 0.003 | -0.83 (-1.34, -0.31) | 0.002 | -0.80 (-1.31, -0.29) | 0.003 |
| FEF_25_-_75_ z-score | -1.13 (-1.56, -0.70) | -0.80 (-1.31, -0.29) | 0.003 | -0.82 (-1.33, -0.30) | 0.002 | -1.03 (-1.53, -0.53) | <0.001 |
| PEF z-score | -0.75 (-1.13, -0.38) | -0.73 (-1.21, -0.25) | 0.003 | -0.72 (-1.21, -0.24) | 0.004 | -0.93 (-1.36, -0.49) | <0.001 |
| DLCOc z-score | -0.47 (-0.94, 0.01) | 0.11 (-0.47, 0.70) | 0.705 | 0.13 (-0.46, 0.72) | 0.668 | -0.01 (-0.55, 0.53) | 0.966 |
| DLCOc/VA | -0.09 (-0.16, -0.01) | -0.05 (-0.14, 0.05) | 0.347 | -0.05 (-0.15, 0.05) | 0.309 | -0.05 (-0.15, 0.05) | 0.323 |
| TLC_pleth_ z-score | 0.40 (-0.02, 0.82) | 0.51 (-0.02, 1.04) | 0.063 | 0.53 (-0.01, 1.07) | 0.057 | 0.56 (0.05, 1.07) | 0.036 |
| FRC_pleth_ z-score | 0.86 (0.39, 1.32) | 0.78 (0.18, 1.37) | 0.011 | 0.79 (0.19, 1.38) | 0.011 | 1.07 (0.50, 1.64) | <0.001 |
| FRC_He_ z-score | 0.14 (-0.38, 0.66) | 0.26 (-0.42, 0.93) | 0.456 | 0.23 (-0.44, 0.90) | 0.504 | 0.33 (-0.31, 0.98) | 0.310 |
| RV_pleth_ z-score | 0.94 (0.48, 1.40) | 0.90 (0.32, 1.48) | 0.003 | 0.90 (0.33, 1.48) | 0.003 | 1.05 (0.45, 1.65) | 0.001 |
| VC_max_ | 0.07 (-0.31, 0.45) | -0.10 (-0.46, 0.25) | 0.572 | -0.08 (-0.43, 0.27) | 0.658 | -0.11 (-0.61, 0.38) | 0.652 |
| LCI_1_ | 0.80 (0.18, 1.41) | 0.66 (-0.11, 1.43) | 0.095 | 0.57 (-0.20, 1.35) | 0.152 | 0.84 (0.06, 1.62) | 0.038 |
| R_5Hz_ z-score | 0.21 (-0.20, 0.61) | 0.33 (-0.17, 0.83) | 0.195 | 0.36 (-0.14, 0.86) | 0.166 | 0.48 (-0.05, 1.01) | 0.078 |
| R_20Hz_ z-score | -0.10 (-0.45, 0.26) | 0.10 (-0.34, 0.55) | 0.650 | 0.14 (-0.31, 0.59) | 0.539 | 0.20 (-0.24, 0.64) | 0.385 |

* adjusted for sex, mother’s ethnic group race, birthweight, gestational age, oxygen dependency at 365wk and Apgar score at 5 mins, smoking in pregnancy, age at time of follow-up, air leak, ventilation group, and pulmonary haemorrhage

** adjusted for sex, mother’s ethnic group race, birthweight, gestational age, oxygen dependency at 365wk and Apgar score at 5 mins, smoking in pregnancy, age at time of follow-up, air leak, ventilation group, pulmonary haemorrhage, antenatal steroids and surfactant

*** matched using data collected prior to the initiation of steroid use, namely: sex, birth weight, birth weight z-score, gestational age in weeks (in keeping with the original trial’s randomisation strata), smoking in pregnancy, multiple birth, ventilation group and Apgar score at 5 mins

**References**

S1 Johnson AH, Peacock JL, Greenough A, Marlow N, Limb ES, Marston L, et al; United Kingdom Oscillation Study Group. High-frequency oscillatory ventilation for the prevention of chronic lung disease of prematurity. N Engl J Med. 2002;347: 633-642.

S2. Sauzet O, Wright K, Marston L, Brocklehurst P, Peacock JL. Modelling the hierarchical structure in data sets with very small clusters: a simulation study to explore the effect of the proportion of clusters when the outcome is continuous. Stats Med. 2013;32: L1429-L1438.

S3. Peacock JL, Sauzet O, Ewings SM, Kerry SM. Dichotomizing continuous data while retaining statistical power using a distributional approach. Stat Med. 2012;31: 3089-3103.

S4. Sauzet O, Breckenkamp J, Borde T, Brenne E, David M, Razum O. A distributional approach to obtaining adjusted comparison of proportions of a population at risk. Emerg Themes Epidemiol. 2016;13: 8.

S5. Zivanovic S, Peacock J, Alcazar-Paris M, Lo J, Lunt A, Marlow N, et al; United Kingdom Oscillation Study Group. Late outcomes of a randomized trial of high-frequency oscillation in neonates. N Engl J Me.d 2014;370: 1121-1130.
